# Blunt Traumatic Vertebral Artery Injury After Cervical Fracture Dislocation: A Systematic Review of the Literature

**DOI:** 10.7759/cureus.65250

**Published:** 2024-07-24

**Authors:** Konstantinos Zygogiannis, Ioannis S Benetos, Dimitrios Stergios Evangelopoulos, Dimitrios Koulalis, Spyros G Pneumaticos

**Affiliations:** 1 Trauma and Orthopaedics Department, Laiko General Hospital of Athens, Athens, GRC; 2 Orthopaedics Department, KAT Hospital, University of Athens, Athens, GRC; 3 3rd Orthopaedic Department, KAT Hospital, National and Kapodistrian University of Athens School of Medicine, Athens, GRC; 4 Orthopaedics and Traumatology Department, Attikon University Hospital, Athens, GRC; 5 3rd Orthopaedic Department, KAT Hospital, University of Athens, Athens, GRC

**Keywords:** mechanism of injury, incidence and prognosis, “ cervical dislocation”, “cervical fracture”, “vertebral artery injury”

## Abstract

Certain high-energy blunt forces may produce unstable cervical fractures with or without dislocation. In rare cases where the superior facets are dislocated, however showing a significant increase within the last decade, these types of injuries may include vertebral artery entrapment at the involvement level leading to artery dissection or occlusion. This phenomenon is usually seen at the C4-C5 and C5-C6 levels of injury. A systematic review of the literature was performed by examining online databases such as PubMed - NCBI, Web of Science, Cochrane Library, Scopus, and Embase to identify relevant scientific articles. Keywords (MeSH terms) used in the search included cervical spine injuries, cervical spine dislocation, cerebrovascular injury, vertebral artery injury, vertebral artery injury management, and incidence of vertebral artery injury. Initially, 1516 studies were identified as a primary search for screening. After excluding papers that did not fulfill the inclusion criteria, 34 studies were included in this review. Vertebral artery injury consists of a severe complication that could compromise a surgical intervention since the patient’s clinical image may be unrevealing at first. Early diagnosis and correct timing constitute the golden standard for adequate treatment. This systematic review aims to summarize the current evidence for the diagnosis, management, and treatment of blunt traumatic vertebral artery injuries.

## Introduction and background

Historically, blunt vertebral arterial injury (BVI) was uncommon and of relatively little significance. While there have been numerous case reports of BVI causing ischemic or hemorrhagic strokes, most studies concerning vertebral artery injuries (VAIs) have focused on cervical fractures [1]. In a variety of studies, only 4% or less of patients presented with blunt vessel injuries. These patients generally had a favorable outcome and this led to the perception that BVI is not very harmful [2]. This idea was strengthened by smaller studies focusing on blunt vessel injuries following cervical spinal trauma [3,4]. Multiple studies have demonstrated an association between vertebral fractures, particularly those of the cervical spine, and blunt cerebrovascular injuries (BCVIs). At the same time, the incidence may vary from 0.53% to 88% depending on the study and the injury [5]. Additionally, numerous studies have highlighted that fractures in the upper cervical spine (C1-C3), with extension into the transverse foramen, and subluxation are considerably linked with BCVI [6].

Cervical spine injuries can be divided into axial and subaxial and can potentially appear in 2-3% of the total cervical spine blunt trauma [7]. These types of injuries can manifest as a complete facet dislocation to a simple ligament and posterior soft tissue strain and can potentially result in a VAI. It is estimated that two-thirds of all cervical fractures and three-quarters of all dislocations are related to the subaxial area of the cervical spine [7]. The sixth and seventh cervical vertebrae alone comprise 39% of all cervical spine fractures [7]. The most frequent etiologies of cervical spine trauma are traffic vehicle accidents with motorcycles (41%), falls from height (27%), physical assault (15%), sports accidents (8%), and massive objects falling on the head [7]. Facet dislocations and fractures can be either unilateral or bilateral [7,8]. A subluxation of the facet, known as a perched facet, may also occur. Distraction mechanisms, often involving rotatory force, can result in facet joint subluxations accompanied by disruption of the disc or capsule. These injuries are observed as a pathological anatomical asymmetry in the alignment of the facets on lateral radiographs or sagittal cuts of a CT scan.

Although upper (axial) cervical spine trauma is a frequent site of injury, there is relatively little literature on its incidence. C1-C3 cervical spine fractures account for 64% of all cervical trauma in geriatric patients [8]. Patients in their forties constitute the largest group (22.8%) of those with axial cervical spine trauma [8]. Among young adults (18−64 years), the most common cause of upper cervical spine injuries is high-energy hyperextension. In a physiologic-mobile cervical spine, the C4 up to C7 segment is considered the most mobile, and as a result that part is more susceptible to injury. However, in older adults, the degeneration and the associated stiffening of the lower cervical spine (such as diffuse idiopathic skeletal hyperostosis) lead to force translation and dissipation in the more mobile upper cervical spine, resulting in a higher incidence of injuries in this age group. Unintentional falls with an incidence of 28.1−48.7% and motor traffic accidents with an incidence of 34.4−37.6% are the primary reasons for these injuries [9]. C2 fractures occur approximately three times more frequently than C1 fractures, with a more frequent apparition of C2 injuries in the elderly population [7]. Occipital condyle injuries are usually rare, with a variety of reports indicating an appearance percentage of about 0.3−0.7% in trauma patients [10].

## Review

Materials and methods

**Figure 1:**
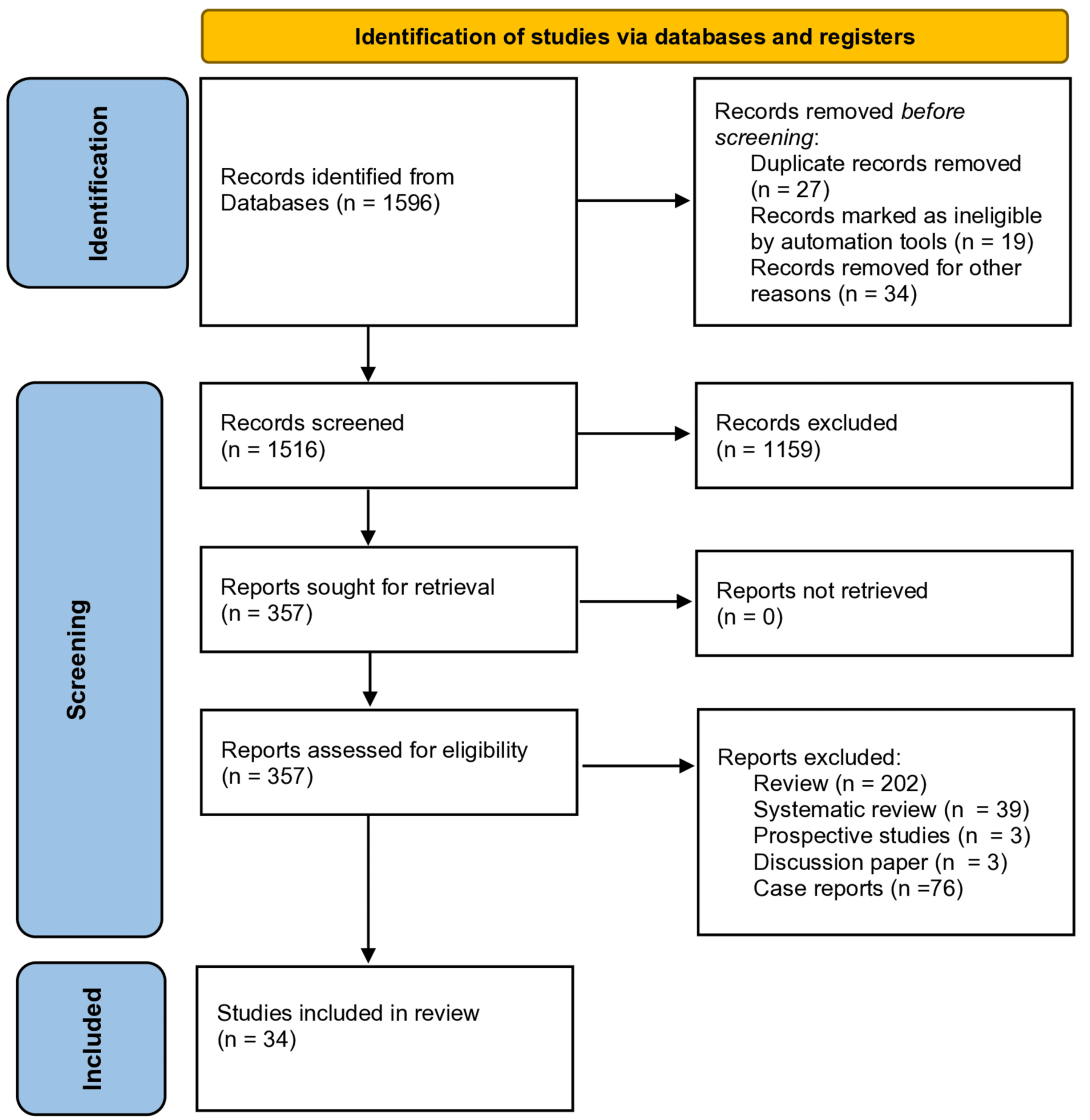
Flowchart based on PRISMA guidelines

A systematic review of the literature was performed based on PRISMA guidelines (Figure 1) by examining online databases such as PubMed - NCBI, Web of Science, Cochrane Library, Scopus, and Embase to identify relevant scientific articles. Keywords (MeSH terms) used in the search included cervical spine injuries, cervical spine dislocation, cerebrovascular injury, VAI, VAI management, and incidence of VAI. An additional search was performed in the archives of medical journals including Spine, European Spine Journal, and Journal of Bone and Joint Surgery. The focus was mainly on studies that involved cervical spine surgery fractures, cerebrovascular injuries, and studies with more than five cases reported. No language or publication date restrictions were applied. Full-text articles were examined to collect additional relevant studies. The studies analyzed the incidence, management, treatment options, and correlation with cervical fractures. A total of 34 articles meeting the specified criteria were included in this comprehensive literature review (Table 1).

**Table 1:** List of included references in blunt vertebral artery injury after cervical spine fracture dislocation.

Authors	Title	Reference
Parent AD et al.	Lateral cervical spine dislocation and vertebral artery injury	[1]
Reid JD and Weigelt JA	Forty-three cases of vertebral artery trauma	[2]
Giacobetti FB et al.	Vertebral artery occlusion associated with cervical spine trauma: a prospective analysis	[3]
Friedman D et al.	Vertebral artery injury after acute cervical spine trauma: rate of occurrence as detected by MR angiography and assessment of clinical consequences	[4]
Mueller CA et al.	Vertebral artery injuries following cervical spine trauma: a prospective observational study	[5]
Lunardini DJ et al.	Vertebral artery injuries in cervical spine surgery	[6]
Nobunaga AI et al.	Recent demographic and injury trends in people served by the Model Spinal Cord Injury Care Systems	[9]
Prasad VS et al.	Characteristics of injuries to the cervical spine and spinal cord in polytrauma patient population: Experience from a regional trauma unit	[10]
Zaveri G and Das G	Management of sub-axial cervical spine injuries	[8]
Vaccaro Alexander R et al.	Update on upper cervical injury classifications: The new AO Upper Cervical Spine Classification System	[7]
Inamasu J and Guiot BH	Vertebral artery injury after blunt cervical trauma: an update	[11]
Willis BK et al.	The incidence of vertebral artery injury after mid cervical spine fracture or subluxation	[12]
Sheppard R et al.	Vertebral artery injury in cervical spine fractures: A cohort study and review of the literature	[13]
Davis JW et al.	Blunt carotid artery dissection: incidence, associated injuries, screening and treatment	[14]
Miller PR et al.	Prospective screening for blunt cerebrovascular injuries: analysis of diagnostic modalities and outcomes	[15]
Cothren CC et al.	Cervical spine fracture patterns predictive of blunt vertebral artery injury	[16]
Berne JD et al.	Helical computed tomographic angiography: an excellent screening test for blunt cerebrovascular injury	[17]
Biffl WL et al.	The devastating potential of blunt vertebral arterial injuries	[18]
Berne JD et al.	The high morbidity of blunt cerebrovascular injury in an unscreened population: more evidence of the need for mandatory screening protocols	[19]
Schippinger G et al.	Injury of the cervical spine associated with carotid and vertebral artery occlusion: case report and literature review	[20]
Simon LV et al.	Vertebral artery injury	[21]
Weller SJ et al.	Detection of vertebral artery injury after cervical spine trauma using magnetic resonance angiography	[22]
Temperley HC et al.	The incidence, characteristics and outcomes of vertebral artery injury associated with cervical spine trauma: A systematic review	[23]
Tokuda K et al.	Anomalous atlantoaxial portions of vertebral and posterior inferior cerebellar arteries	[24]
Zimmerman HB and Farrell WJ	Cervical vertebral erosion caused by vertebral artery tortuosity	[25]
Madawi AA et al.	Radiological and anatomical evaluation of the atlantoaxial transarticular screw fixation technique	[26]
Paramore CG et al.	The anatomic suitability of the C1-2 complex for transarticular screw fixation	[27]
Wei CW et al.	Blunt cerebrovascular injuries: diagnosis and management outcomes	[28]
Hadley MN et al.	Guidelines for the management of acute cervical spine and spinal cord injuries	[29]
Brommeland T et al.	Best practice guidelines for blunt cerebrovascular injury (BCVI)	[30]
Elder T and Tuma F	Bilateral vertebral artery transection following blunt trauma	[31]
Wynn MS et al.	Patient specific predictive factors of vertebral artery injury following blunt cervical spine trauma	[32]
Hadley MN et al	Management of vertebral artery injuries after nonpenetrating cervical trauma	[33]
Nakamura Y et al.	Vertebral artery occlusion associated with blunt traumatic cervical spine injury	[34]

Results

*Incidence* 

The frequency of VAI can vary significantly depending on the accurate diagnosis of asymptomatic patients. A blunt spine injury of significant force, such as in a motor vehicle accident (MVA), aside from a cervical fracture, could potentially provoke extensive soft tissue and VAI (Table 2). Conversely, even a minor mechanism of trauma, like chiropractics, aerobics, or yoga, is recognized to induce VAI, predominantly dissection, without significant cervical spine injury in most cases [11]. Hence, determining the precise incidence of VAI following blunt cervical trauma under these circumstances is challenging. Willis et al. highlighted the incidence of VAI within groups deemed "high-risk": patients who present with cervical spine fractures affecting the transverse foramen and with CT scans that reveal facet joint dislocation/subluxation. For this purpose, they conducted a prospective study using digital subtraction angiography (DSA). Their study group involved 26 patients in which VAI was observed in 12 cases (46%) [12]. In another retrospective study, which was performed on 1,894 computed tomography (CT) reports from patients who had undergone imaging of their cervical spine and/or vertebral arteries within a 12-month timeframe, spanning from June 2018 to June 2019, 68 patients (3.59%) were identified as having a confirmed cervical spine fracture. They spanned ages from 18 to 97 years and comprised 39 males (57.4%) and 29 females (42.6%). Among the 68 patients with confirmed cervical spine fractures, 5 (7.35%) were diagnosed with VAI, all of which were associated with fractures in their upper cervical spine [13].

**Table 2:** Incidence of vertebral artery injury VAI: vertebral artery injury; CSI: cervical spine injury; FDS: facet dislocation/subluxation; NA, not available; TFF: transverse foramen fracture; DSA: digital subtraction angiography; CTA: computed tomography angiography.

Study		No. of patients	Imaging	VAI	VAI/CSI	VAI/FDS	VAI/TFF
Willis et al. (1994) [12]		26	DSA	12	12/26	8/18	7/16
Miller et al. (2002) [15]		241	DSA	43	43/109	12/27	28/58
Cothren et al. (2003) [16]		605	DSA	92	92/290	N/A	N/A
Berne et al. (2004) [17]		486	CTA	14	14/13	N/A	N/A

The latest data regarding the incidence of VAI originated from various level I trauma centers where extensive prospective angiographic studies were conducted. The incidence of VAI injury among all patients who presented with blunt trauma varied between 0.20% and 0.77% [14-16]. Berne et al. identified 14 patients (0.44%) with VAI out of 3150 total admissions for blunt trauma, utilizing computed tomography angiography (CTA) [17]. Miller et al. adopted an assertive strategy by conducting DSA in nearly all blunt trauma patients who met their screening criteria. Among the 216 patients meeting the criteria, they identified 49 (22.7%) cases of VAI. The overall incidence of VAI among all admissions for blunt trauma was 0.71% [15]. Biffl et al. suggested results similar to those of Miller et al. but on a larger scale. Among 605 patients meeting their DSA screening criteria, they identified 92 cases (15.2%) of VAI, using criteria similar to Miller et al. However, their screening criteria were more inclusive. The incidence of VAI among a total of 12,552 admissions for blunt trauma was 0.77% [18]. Berne et al. reported 14 patients (0.44%) with VAI among 3150 total admissions for blunt trauma utilizing CTA [19]. These studies conducted at large trauma centers had more comprehensive screening criteria for DSA or CTA compared to earlier studies conducted by spine surgeons. They intended to screen not only for VAI but also for carotid artery injury (CAI). Occasional reports have indicated a coexistence of VAI and CAI in the same patient [16,20].

Mechanism of Injury and Fracture Patterns

Several spinal injuries and fracture patterns have been highlighted as a cause of blunt VAI. Fractures involving the transverse foramen, a corridor via which the vertebral artery (VA) travels from C6 to C1, presented a high incidence of VAI [21,22]. According to Muellier et al., the incidence of VAI in cervical spine subluxations can be as high as 31% [5]. A review paper by AO Spine states that traffic accidents with motor vehicles were the most common cause of injury in seven out of nine studies, while falls were predominant in two out of nine studies. Overall, among the studies analyzing the mechanism of injury (MOI) of VAI, 139 out of 240 cases (57.9%) were attributed to MVAs, and 61 out of 240 cases (25.4%) were due to falls. Other less frequent causes included pedestrians being struck by vehicles, falls from heights, snow-sport accidents, horse riding accidents, hangings, diving accidents, and miscellaneous causes. Seven out of the remaining 24 studies, which reported incidental cases of VAI, did not provide information on the MOI [23].

Anatomical Variations

In the case of an anatomical variation of the VA, a potential injury can occur intraoperatively or at the moment of injury, even in simple fracture patterns [24,25]. In the region where the atlas (C1) and axis (C2) vertebrae meet, the incidence of VA anomalies is reported as high as 2.3% [24]. However, recent radiological and anatomical investigations of this area suggest that up to 20% of individuals may exhibit abnormal positioning of the VA [26]. Paramore et al. discovered that 18% of subjects displayed a C2 transverse foramen situated unusually high on at least one side, posing a significant risk to the VA [27]. Other abnormalities include the course of the VA below the superior portion of the articular surface of the axis, which may be abnormally medial, posterior, or raised, potentially creating a very restricted C2 isthmus. Furthermore, Madawi et al. revealed that in 20% of cases, the inferior surface of the C2 lateral mass showed grooves due to the VA passage, which caused the pedicle to erode and the lateral mass to shrink. The development of bony subluxation in the cervical vertebrae can significantly alter both the bone and vascular anatomy, making the artery vulnerable to damage even with accurate screw insertion [27]. As a result, this elevates the risk of VA injury.

Selection Criteria and Imaging

Biffl et al. suggested the Denver Screening Criteria for Blunt Cerebrovascular Injury. Nevertheless, these screening criteria also encompass CAI, which is a completely different entity from VAI in terms of clinical image, prognosis, and management [18]. Consequently, additional research is necessary to establish distinct screening criteria tailored explicitly to VAI. VA screening may be required based on the patient’s symptoms and fracture pattern. The most commonly reported symptoms among various studies included headaches, clinical signs suggesting vertebrobasilar ischemia, and vertigo, and the percentage of symptoms apparition or a statistical analysis was not available from the studies. The Denver grading scale proposes four distinct categories to classify the severity of injury based on the imagistic findings and not the clinical image. Grade 1 includes an intimal irregularity with <25% narrowing, in Grade 2 the patient presents findings compatible with dissection or intramural hematoma with >25% narrowing, in Grade 3 the imagistic findings suggest pseudoaneurysm while in Grade 4 there is occlusion and in Grade 5 transection of the vessel with extravasation of contrast. CTA has replaced DSA as a golden standard tool in the diagnosis of VAI due to its reported accuracy in several studies to detect clinically significant injuries [28]. Magnetic resonance angiography (MRA) is another non-invasive tool that can be used in the screening of these types of injuries. In comparison with the other imagistic options, MRA can diagnose at the same time soft-tissue injuries that include vertebral discs or ligaments or spinal cord pathology [29]. Additionally, several unblinded prospective studies comparing DSA with MRA suggest that their sensitivity has a wide range of variation and can be between 50% and 100% while the specificity can vary between 29% and 100% [5]. Finally, in intubated polytrauma patients, since the MRA is riskier to perform due to the time that it is demanded for the patient to undergo scanning, CTA should be performed as it is safer for the patient at risk of VAI (Table 3) [15].

**Table 3:** Sensitivity and specificity of CTA versus MRA based on the study findings of Miller et al. 2002 [15]. CTA: computed tomography angiography; MRA: magnetic resonance angiography; VAI: vertebral artery injury.

Sensitivity and specificity rates of CTA versus MRA in diagnosing VAI		
-	Computer tomographic angiography	Magnetic resonance angiography
Sensitivity	53%	47%
Specificity	99%	97%
Comments	The golden standard in diagnosis	It should be avoided if the patient is unstable but it offers additional information for soft-tissue and spinal cord injuries

Management

Patients presenting with symptomatology compatible with acute ischemic stroke due to blunt VAI, without associated injuries or contraindications like active hemorrhage, should be considered for systemic thrombolytic therapy (using tissue-type plasminogen activator) if they seek treatment within 3 hours of symptom onset. For patients who present within 4.5 hours from the moment of injury or who are not suitable for systemic thrombolytic therapy, catheter-directed thrombolysis could be considered a viable solution. Patients who do not meet the criteria for thrombolytic treatment can be addressed with antiplatelet therapy, anticoagulation, endovascular intervention, or open operative repair depending on the severity of the injury [30]. Based on the grading scale, injuries that concern Grade 1 and 2 imagistic findings can be treated with a single antiplatelet agent (aspirin 81 mg or 325 mg). Findings compatible with Grade 3 injury require dual antiplatelets or therapeutic anticoagulation (heparin drip with a partial thromboplastin time (PTT) goal of 60 to 90), while injuries compatible with Grade 4 VAI necessitate dual antiplatelets or therapeutic anticoagulation used on a case-by-case basis with input from neurosurgery (Table 4) [18].

**Table 4:** Proposed treatment of vertebral artery injury based on Denver Grading Scale PTT: partial thromboplastin time.

Denver BCVI Grading Scale With Proposed Treatment According to Grade			
Grade 1	Intimal irregularity with <25% narrowing	Conservative	Single antiplatelet agent (aspirin 81 mg or 325 mg)
Grade 2	Dissection or intramural hematoma with >25% narrowing	Conservative	Single antiplatelet agent (aspirin 81 mg or 325 mg)
Grade 3	Pseudoaneurysm	Conservative	Dual antiplatelets or therapeutic anticoagulation (heparin drip with a PTT goal of 60 to 90)
Grade 4	Occlusion	Conservative or surgical	Dual antiplatelets or therapeutic anticoagulation used on a case-by-case basis with input from neurosurgery/internal trapping or stent-assisted coiling
Grade 5	Transection with extravasation of contrast	Surgical	Internal trapping or stent-assisted coiling

An optimal therapy can vary between anticoagulation and antiplatelet medication for managing symptomatic patients while always taking into consideration the bleeding risk, the anatomic location of the lesion, and the severity of the injury. Endovascular therapy and operative repair are meant for patients with higher-grade lesions or patients whose injuries are more likely to progress. There are currently no widely approved protocols to decrease the risk of neurologic events or recurrent dissection in patients with known VAIs. However, patients are instructed to avoid contact sports or any activity involving abrupt neck movements for a minimum period of six months. Additionally, controlling hypertension and avoiding estrogen-containing medications can prove positive measures [31].

Discussion

In the current literature, VAI after blunt cervical trauma is considered a rare entity. However, the most common cause of blunt trauma, which is likely to lead to VAI, is MVAs [7]. What is crucial to highlight is that a significant amount of VAI is left undiagnosed, as the majority of patients present no symptomatology. Even screening methods have been suggested in order to eliminate the chance of any undiagnosed asymptomatic cases of VAI, as presented by Wynn et al. in 2021 [32]. This explains the necessity of high awareness among spine surgeons regarding VAI. Controversy, however, still exists regarding the proper imaging tool as well as the effective management, especially when spinal cord injury is present and early surgery is essential for recovery. More specifically, patients suffering from transverse foramen fractures, as well as facet dislocations or subluxations are at the highest risk of VAI, as described by Willis et al. [12]. Cothren et al., in their study, presented that the majority of VAI was co-existent with transverse foramen fractures and facet dislocation/subluxation in 55% and 26%, respectively [16]. More recent studies describe an incidence of 0.20-0.77% regarding VAI in major trauma centers [14]. Thus, the keystone in VAI management is the proper and detailed diagnosis via diagnostic modalities that are available in major trauma centers. Despite the efficacy of MRA, CTA, and duplex ultrasound scanning (DUS), DSA is the most successful diagnostic tool, especially when performed as early as possible post-trauma, even in 72 hours. Regarding the most frequent types of injury presented in the current literature, dissection and occlusion are highlighted in most cases [11].

Furthermore, there is an excellent debate in the current literature regarding the proper management of VAI, as the mechanism and type of injury suggest the appropriate management actions. Many treatment options are available, including the embolization of the affected VA in the cases of life-threatening transection of the VA, as well as endovascular thrombolysis for cases with rapid neurological deterioration after vertebrobasilar stroke where embolectomy is also likely to be used as a treatment method. Special consideration is given in the cases of pseudoaneurysm of VA, where the treatment includes either open methods or stent placement, aiming to decrease the occlusion deterioration. However, when stroke is not present, non-invasive agents have also been used, mainly consisting of anticoagulants such as heparin or antiplatelet medicines, primarily aspirin, which can be used in even asymptomatic patients [11,33]. Last, but not least, the safety of the use of the anticoagulant agents mentioned above when SCI is present is not yet thoroughly investigated in the current studies, as well as the proper algorithm of surgical management when an incomplete SCI is present together with a VAI, as prospective and even retrospective studies including patients with such both entities are limited. Nakamura et al., in 2021, however, proposed the endovascular embolization of the VA occlusion before stabilizing the cervical spine injury with instrumentation to eliminate the stroke incidence [34]. The limitation of this study includes the risk of bias where the methodological process may induce inaccuracies and the potential overlook of important information due to the volume of information.

Future considerations and implications

Since VAI complications can range from asymptomatic to more serious clinical entities including neurologic deficit, pseudoaneurysm, infarction, late-onset hemorrhage, and death, a detailed imagistic selection must be made in case of suspicion. Statistical analysis regarding the percentage of symptoms at the moment of arrival at the emergency department of patients with VAI was not available. A variety of studies suggest that in some cases such an injury may be unrevealing at first or during the whole hospital stay. As a result, a possible standard protocol of investigation based on specific fracture patterns, even in asymptomatic patients, should be adopted from all trauma centers. The available data may have a wide range of variations depending on the percentages of traffic accidents and the frequency of criminal acts in each country. Factors that may directly affect the statistical outcome are the geographic area, the sample size, and the location of each hospital. Self-reported data could also potentially affect the statistical outcome. As the literature lacks evidence from large sample sizes or multicenter studies, further research with long-term follow-up should be made to assess the quality of the current protocols.

## Conclusions

Depending on the study, VAI after cervical fracture dislocation can be referred to as a less or more frequent entity. Early diagnosis and management can offer a significantly favorable outcome compared with the cases where the diagnosis slipped. VAI can be treated successfully conservatively or surgically depending on the grading scale but in the case of a cervical fracture, it can compromise an early intervention. A potential cervical spine intervention in cases where VAI could be a contraindication can prove devastating for the patient. A variety of imagistic tools are available for a proper diagnosis that can be used depending on the availability and the patient’s condition. Finally, a multidisciplinary approach is necessary to accurately address these patients.
